# [Retracted] Upregulation of Stat1‑HDAC4 confers resistance to etoposide through enhanced multidrug resistance 1 expression in human A549 lung cancer cells

**DOI:** 10.3892/mmr.2025.13461

**Published:** 2025-02-17

**Authors:** Chutima Kaewpiboon, Ratakorn Srisuttee, Waraporn Malilas, Jeong Moon, Sangtaek Oh, Hye Gwang Jeong, Randal N. Johnston, Wanchai Assavalapsakul, Young-Hwa Chung

Mol Med Rep 11: 2315–2321, 2015; DOI: 10.3892/mmr.2014.2949

Following the publication of this article, an interested reader drew to the Editor's attention that a pair of data panels in the cellular images shown in Fig. 1A on p. 2317 appeared to be overlapping, such that data which were intended to show the results from differently performed experiments had appparently been derived from the same original source. Moreover, in Fig. 1C and 5A, there appeared to be discrete splicing events for gel slices featured in these figure parts, and for the immunoblotting experiments shown in Fig. 2B, striking likenesses of certain of the bands appeared in different positions in different gels relative to each other where different experimental conditions were being shown, which seemed difficult to attribute purely to coincidence.

After having conducted an internal investigation, the Editor of *Molecular Medicine Reports* agrees with the reader that there were anomalies associated with the presentation of Figs. 1, 2 and 5. Therefore, on the grounds of a lack of confidence in the presented data, the Editor has decided that the article should be retracted from the publication. Upon contacting the authors, all the authors agreed with the decision to retract this paper, except for Dr Randal Johnston, who was not contactable owing to his retirement several years ago. The Editor apologizes to the readership for any inconvenience caused, and we also thank the reader for bringing this matter to our attention.

